# On the Use of Strain Path Independent Metrics and Critical Distance Rule for Predicting Failure of AA7075-O Stretch-Bend Sheets

**DOI:** 10.3390/ma13173660

**Published:** 2020-08-19

**Authors:** Andrés Jesús Martínez-Donaire, Domingo Morales-Palma, Carpóforo Vallellano

**Affiliations:** Department of Mechanical and Manufacturing Engineering, University of Seville, Camino de los Descubrimientos s/n, 41092 Sevilla, Spain; dmpalma@us.es (D.M.-P.); carpofor@us.es (C.V.)

**Keywords:** formability limits, failure prediction, stretch-bend forming, critical distance rule (CDR), strain path independent metrics, AA7075-O, finite element analysis (FEA)

## Abstract

The strain-based forming limit curve is the traditional tool to assess the formability of metal sheets. However, its application should be restricted to proportional loading processes under uniform strain conditions. Several works have focused on overcoming this limitation to characterize the safe process windows in industrial stretch-bend forming processes. In this paper, the use of critical distance rule and two path-independent stress-based metrics are explored to numerically predict failure of AA7075-O stretch-bend sheets with 1.6 mm thickness. Formability limits of the material were experimentally obtained by means of a series of Nakazima and stretch-bending tests at different thickness-over-radius ratios for inducing controlled non-uniform strain distributions across the sheet thickness. By using a 3D calibrated finite element model, the strain-based forming limit curve was numerically transformed into the path-independent stress and equivalent plastic strain polar spaces. The numerical predictions of necking strains in the stretch-bending simulations using the above approaches were successfully compared and critically discussed with the experimental results for different values of the critical distance. It was found that failure was triggered by a critical material volume of around the half thickness, measured from the inner surface, for the both path-independent metrics analyzed.

## 1. Introduction

Conventional sheet metal operations of ductile materials are mainly limited by the appearance of localized necking and the subsequent ductile fracture. This failure mechanism begins with the strain localization in a narrow band that generates a neck, which evolves unstably until material fracture. In operations such as stamping, deep drawing, or stretch-bend forming, the material is deformed over shaped punches or dies with small curvature radii, inducing both a non-uniform strain distribution across the thickness due to the bending effect and also complex strain paths [1]. In this context, a precise characterization of forming limits at necking is of great importance for setting the safe process windows in complex industrial processes, especially in highly competitive sectors, such as the aeronautical or automotive industries.

The widely used forming limit diagram (FLD), introduced by Keeler and Backhofen [2], establishes a boundary between the principal strain states that allows the safe forming of the sheet and those that cause failure by necking. This boundary is known as the forming limit curve (FLC) and it is obtained under proportional loading and uniform strain conditions in Nakazima- and/or Marciniak-type tests in laboratory. However, despite the existence of recent active research devoted to detecting the onset of plastic instability and focused on the FLC determination [3,4,5,6,7,8,9,10], this curve has demonstrated to be very sensitive to the strain-path effect [11]. Moreover, the formability limits triggered by the FLC does not account the bending effect because its determination was conducted under almost in-plane stretching, that is, uniform strain conditions, by using punches with a gentle curvature or in absence of it [3]. Both facts point out the limitations of the FLC for evaluating failure in common industrial processes [12].

The beneficial role of the punch curvature on the sheet failure has been extensively studied in the past, pointing out that formability limits under a stretch-bend deformation mode are higher than in stretching tests subjected to uniform strain conditions [13,14,15,16,17,18,19]. From an experimental approach, Charpentier [13] revealed an enhancement of more than 50% in the limit strains when using an elliptical punch of 24 mm radius compared to a 95 mm punch radius over low carbon steel sheets of 1.85 mm thickness. More recently, differences in the FLC obtained by using Nakazima- and Marciniak-type tests for dual-phase (DP) steel and aluminum sheets have been pointed out by Merklein et al. [17] and explained by the different punch curvature and the strain path induced by them.

Within an analytical framework for predicting formability in presence of strain gradients, Tharret and Stoughton [20] found that necking at the convex surface (outer face) of stretch-bend mild steel sheets appeared when the strain on the concave side (inner surface) achieved a value consistent with the forming limit under in-plane stretching, i.e., the FLC. This criterion, known in the literature as the concave side rule (CSR), as well as the traditional mid-plane rule (MPR), which characterizes the material formability by using the mean value through the sheet thickness, may provide inaccurate predictions depending on the bending severity [18,21,22,23]. Wu et al. [24] developed a failure approach based on the bending-modified FLC (BFLC) to account for the stretch-bending condition, by making use of the stretch bendability index (SBI) concept, previously introduced by Sadagopan [25]. Its application using finite element analysis (FEA) simulations to angular stretch bend tests (ASBT) over DP steel yielded reasonably good predictions in terms of failure height. More recently, Neuhaser et al. [19] extended the Keeler–Brazier FLD by a third axis, the superimposed bending dimension, and pointed out that the more severe the bending, the higher the formability of the DP600 steel sheets studied. Vallellano et al. [26] analyzed the failure mechanisms expected in a sheet subjected to a non-uniform strain distribution: a necking-controlled failure, which occurs when the less strained material layers in the sheet thickness (inner layers in terms of curvature) reach a plastic instability condition, meaning that the entire sheet thickness becomes plastically instable and will neck; and a fracture-controlled failure, which arises when the most strained material layers (outer layers in terms of curvature) reach their strength limit and get fracture. Thus, they introduced and explored the critical distance rule (CDR), which is based on an extension of the critical distance concepts traditionally used for analyzing the fatigue and fracture behavior of notched members [27], to predict the material formability under non-uniform strain/stress distributions [28,29,30]. This rule suggested that failures by necking or by fracture are triggered by the development of damage in a certain material volume, characterized by a critical distance, located at the inner or outer zone of the sheet thickness, respectively.

On the other hand, it is well known that changing strain paths during the deformation process modifies the shape and location of the strain-based FLC [11,31]. Because of the non-proportional deformation history inherent to the stretch-bending processes [1,17,28], the formability limits need to be assessed in terms of strain path independent metrics. In this regard, different stress-based metrics, which need to assume a constitutive model of the material, i.e., plastic flow curve, yield criterion, and hardening model, have demonstrated to be less sensitive to strain path history than the strain-based FLC. Particularly, from its conceptualization by Arrieux [32,33] and Stoughton [34], the forming limit stress curve (FLSC) within the principal stress space, has been extensively investigated in the last few years [35,36,37,38,39]. Panich et al. [37] derived the fracture forming limit stress curves (FFLSC), by using Hill’48 and Yld’89 plasticity models, from the strain-based fracture forming limit curve (FFLC) obtained by using the Nakazima test over an advanced high strength steel grade 980. They applied both metrics for predicting fracture in three experiments, namely, rectangular cup drawing, diabolo, and mini-tunnel forming tests. They concluded that the stress-based metrics, i.e., the FFLSC, could more precisely describe the failure than the FFLC. It is worthy to note that other stress-based metrics, which take into account other variables, have been proposed claiming also their independency with complex strain-paths [40,41,42]. Simha et al. [41] proposed the extended stress-based forming limit curve (XSFLC), within the mean stress versus equivalent stress space, for predicting the onset of necking during tubular hydroforming of DP600 steel under non-proportional loading paths. Stoughton and Yoon [42] suggested transforming the experimental FLC into the equivalent plastic strain-based forming limit curve (epFLC). This path-independent curve was plotted within the polar space, $\varepsilon_{eq}^{p}\sin\left(\theta \right)\;\mathrm{versus}\;\varepsilon_{eq}^{p}\cos\left(\theta \right)$, where θ represents the angle of the local strain ratio ($\beta =d\varepsilon_{2}/d\varepsilon_{1})$. Nguyen et al. [43] found that its application for predicting failure in AA6063 tube hydroforming operations under non-proportional strain paths yielded good results. However, most of the application of these metrics has been focused mainly on processes under nearly uniform deformation conditions, not taking into account the bending effect.

The success in predicting sheet failure by FEA during the design stages of the manufacturing of industrial parts subjected to complex stretch-bend forming conditions need to simultaneously account both the effect of non-uniform strain distributions across the sheet thickness and the existence of complex strain paths. This work revisits two failure criteria based on the critical distance rule (CDR) along with two path-independent stress-based metrics, namely FLSC and epFLC, for assessing failure of AA7075-O stretch-bend sheets with 1.6 mm thickness. The formability limits of the material were experimentally obtained by means of a series of Nakazima and stretch-bending tests at different bending ratios (*t*_0_/*R*) for inducing controlled non-uniform strain conditions across the sheet thickness. By using a 3D calibrated finite element model, the strain-based FLC was numerically transformed into the path-independent stress and equivalent plastic strain polar spaces, respectively. The numerical predictions of necking strains in the stretch-bending simulations over cylindrical punches of different diameters by using both failure approaches were successfully compared and critically discussed with the experimental results, for different values of the critical distance. It was found that for AA7075-O sheets of 1.6 mm thickness, failure in stretch-bending was triggered by a critical material volume of around the half thickness, $d_{crit}=0.42-0.5t_{0}$, measured from the inner surface, for the both path-independent metrics analyzed.

## 2. Experimentation

The investigation was performed over AA7075-O sheet with 1.6 mm thickness, which is extensively used in the aeronautical industry. The tensile properties were obtained according to the standards ASTM E8/E8M-08 [44] and ASTM E132-04 [45] and the plastic anisotropy coefficients (*r*) were evaluated using ASTM E517-00 [46]. Regarding the formability limits, the conventional forming limit curve (FLC) at necking was obtained using Nakazima tests (stretching tests). In addition, a series of stretch-bending tests using cylindrical punches of different diameters, ranging from ϕ1 up to ϕ20 mm, were carried out.

In the experiments, the commercial strain imaging systems Vic 2D^®^ (Correlated Solutions, Columbia, SC, USA) and ARAMIS^®^ (GOM, Germany), based on digital image correlation (DIC) technique [47,48,49], were used to measure the principal strain history at the outer surface of the tested specimens. It is worthy to note that DIC techniques are being increasingly and extensively used to automatically measure strain maps at the whole surface with high accuracy in different applications, such as, thermal deformation of materials [50], metal forming [51], mechanical testing of building materials [52], or biomechanical analysis of bone tissues [53], among others.

### 2.1. Tensile Tests

The tensile experiments were carried out on a universal testing machine (model 810, MTS, Eden Prairie, MN, USA) at room temperature. The specimens were cut in three different orientations: 0°, 45° and 90° from the rolling direction. At least three specimens were tested in each direction. The longitudinal and the transverse plastic true strains were measured using a 2D optical strain measurement system Vic 2D^®^, based on the DIC technique. To this end, a stochastic speckled pattern was applied at the outer surface of the specimens by spraying a fine cloud of black paint over a thick layer of matte white paint. A 5 MPx digital CCD camera (LIMESS, Krefeld, Germany) continuously captured images during the deformation process at a rate of five frames per second.

**Figure 1:**
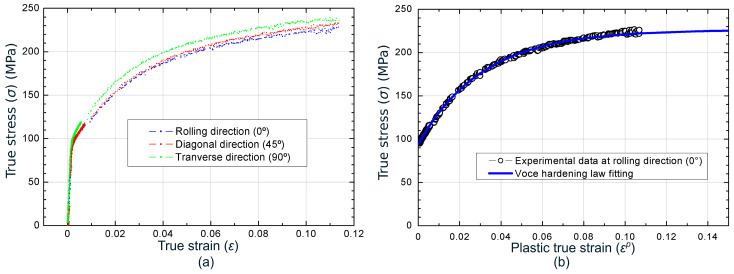
(**a**) True stress vs. true strain at rolling, diagonal and transverse directions; (**b**) experimental data and fittings of true stress vs. plastic true strain at rolling direction.

Figure 1a depicts the experimental true stress vs. true strain curves obtained up to the onset of necking for the three orientations. The mean values of the Young’s modulus (*E*), yield stress (*σ*_*Y*, 0.2%_), ultimate tensile strength (*UTS*), and Lankford’s coefficients (*r*) of the metal sheet for each testing direction are given in Table 1. A Poisson coefficient (ν) of 0.3 was assumed for the FEA in the present work.

The plastic behavior at rolling direction was fitted using a Voce-type hardening law (Figure 1b), which allowed successfully modelling the experimental data measured during the formability tests, as will be shown later. This fact is in agreement with the works by Jain et al. [54], Butuc et al. [55], and Li et al. [56], in which good predictions of limit strains for aluminum alloys were obtained when the stress-strain data from uniaxial tests were fitted to a Voce equation. The Voce expression for the material here analyzed at 0° is given by:

(1) $$\sigma_{eq}\left(\mathrm{MPa}\right)=226.3-131.9e^{-32.23\varepsilon_{eq}^{p}}$$

where $\sigma_{eq}$ is the equivalent stress and $\varepsilon_{eq}^{p}$ represents the equivalent plastic strain.

### 2.2. Nakazima Tests

The conventional FLC at necking was obtained by means of Nakazima tests (hemispherical ϕ100 mm punch) performed using a universal sheet metal testing machine (model 142-20, Erichsen, Germany) at room temperature and following the testing conditions of the standard ISO 12004-2:2008 [3]. The tribological system was a combination of Vaseline and Polytetrafluoroethylene (PTFE). A 3D optical deformation measurement system, ARAMIS^®^, based on the DIC technique, was used for evaluating the strain history at the outer surface of the specimens. Test images were recorded by means of two 1.3 MPx CCD cameras at 10 frames per second.

**Figure 2:**
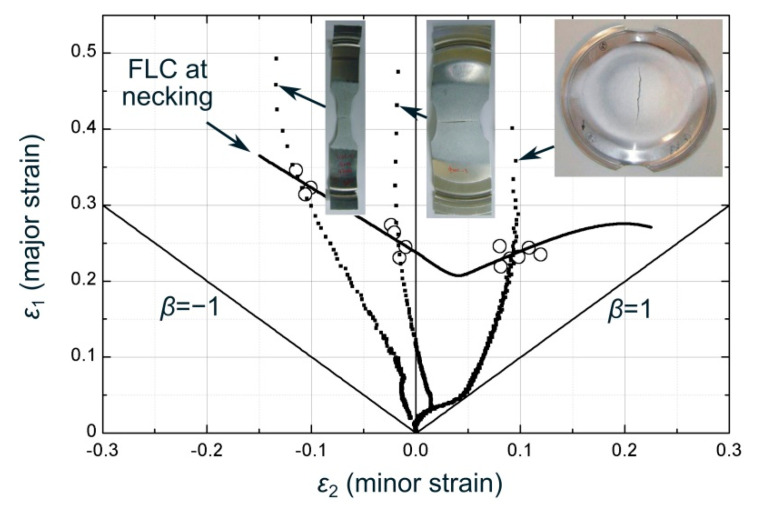
Forming limit curve for AA7075-O sheets of 1.6 mm thickness and strain paths from DIC.

The FLC determination was done in accordance with a time-dependent methodology (t-d method) developed by the authors [57,58], which provided almost identical results to those obtained by the standardized ISO 12004-2 method. A detailed description of the physical basis of the t-d method and a comparative analysis of the results can be found in [57,58,59,60]. Figure 2 depicts the predicted FLC at necking for the AA7075-O sheets, the tested specimen geometries and the experimental strain paths during the experiments.

### 2.3. Stretch-Bending Tests

**Figure 3:**
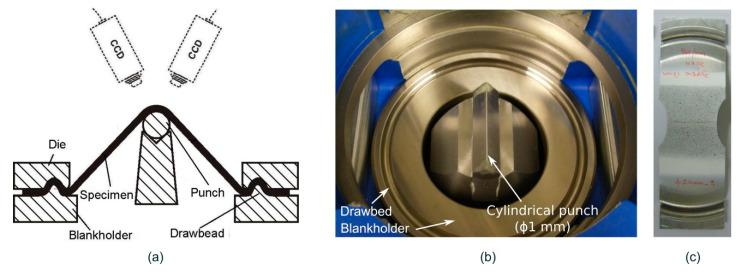
(**a**) Schematic and (**b**) experimental setup of stretch-bending tests; (**c**) geometry of the tested stretch-bend specimens.

In these tests, the specimens were deformed over cylindrical punches of different diameters, ϕ20, ϕ10, ϕ5, ϕ3 and ϕ1 mm, inducing an increasing strain gradient through the sheet thickness due to the bending generated by the curvature of the punches. Figure 3 shows an illustrative scheme of the elements involved, the experimental setup of the stretch-bending tests using a ϕ1 mm punch and the geometry of the tested specimens.

**Figure 4:**
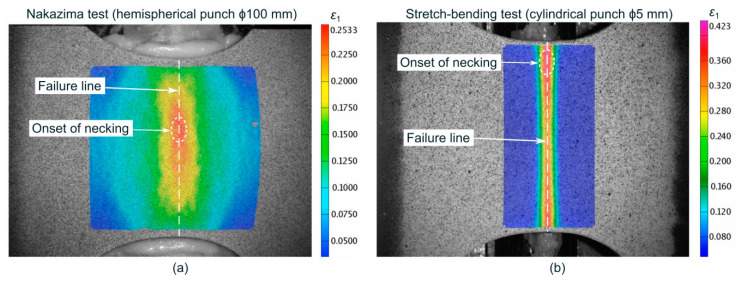
Experimental major strain contours at the outer surface in a stage near the onset of necking for a (**a**) ϕ100 mm Nakazima punch and (**b**) ϕ5 mm cylindrical punch.

The experiments exhibited a strain state close to plane strain conditions according to the specimen geometry selected. Three specimens, at least, were tested for each punch diameter. The testing parameters were identical to those in Nakazima tests and the experimental limit strains at the onset of necking were estimated using the previously mentioned t-d method developed by the authors [57,58]. For every sample, as before, the forming limits were evaluated in three sections perpendicular to the fracture, according to the recommendations of standard ISO 12004-2 [3]. It should be noted that the failure mechanism was always necking followed by ductile fracture both in the Nakazima and stretch-bending tests. In this regard, Figure 4 depicts the major strain contours at the outer surface of the specimens in a stage near the onset of necking for experiments using ϕ100 mm (Nakazima) and ϕ5 mm punches. It is worthy to note that the strain localization and, thus, the onset of failure was located about half the width of the sheet for the Nakazima test whereas it was shifted close to the free-edge for the 5mm punch. The trend for the rest of cylindrical punches is detailed in Section 3.1 along with the numerical predictions for a couple of cylindrical punches.

**Figure 5:**
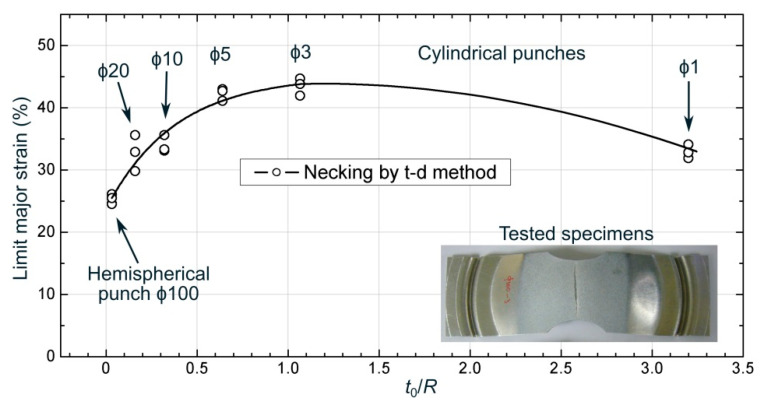
Limit major strain at necking versus *t*_0_/*R* ratio near plane strain conditions obtained using the t-d method.

**Figure 6:**
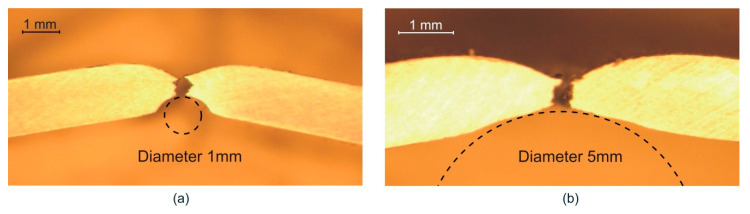
Cross section of AA7075-O specimens after fracture using cylindrical punches of (**a**) ϕ1 mm and (**b**) ϕ5 mm.

Figure 5 plots the mean value of the limit major strain at necking versus the bending ratio (*t*_0_/*R*) for each tested specimen. As can be seen, the predictions of the necking strains exhibited an upward trend with increasing *t*_0_/*R* ratio, up to the ϕ3 mm cylindrical punch, because of an increasing bending effect [13]. However, the limit strains dropped drastically for the ϕ1 mm punch. The reason for this behavior can be found by analyzing locally the contact area between the punch and the sheet. As shown in Figure 6a, the inner surface of the sheet was locally indented by the ϕ1 mm punch due to the severe normal stresses generated in the punch-sheet contact. This indentation at the inner zone surface modifies severely the strains at the outer zone, reducing them, and diminishing the apparent beneficial effect of bending. This phenomenon was not observed for the larger diameter punches, i.e., from ϕ20 up to ϕ3 mm, as can be seen in Figure 6b for a ϕ5 mm cylindrical punch. A similar trend was also observed by the authors in previous works over high-strength steels sheets [30].

In summary, the stretch-bending limit strains increased with increasing strain gradients measured by means of *t*_0_/*R* bending ratio, except for the smallest punch diameter. The results allowed quantifying an enhanced formability at necking of around 72% greater when comparing a cylindrical ϕ3 mm punch versus a ϕ100 mm punch.

## 3. Numerical Modelling

**Figure 7:**
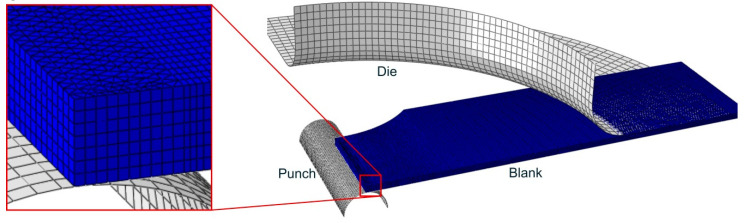
Virtual setup modelled and finite element mesh (one quarter model).

Simulations of the stretching and stretch-bending tests were carried out in ABAQUS/Standard (Dassault Systèmes, France) using a mesh of 3D deformable solid elements for the metal sheet and rigid 2D elements for the punch and die. Figure 7 shows the virtual setup considered in one of the simulations with cylindrical punch.

A one quarter model was simulated due to the symmetry of the problem. Several layers through thickness, depending on the bending severity, were arranged in order to adequately represent the 3D stress/strain states around the failure zone and across the sheet thickness. A mixed of wedge (C3D6H, hybrid formulation) and brick (C3D8R, reduced integration scheme and enhanced hourglass control) elements were used, based on previous research by the authors [22,61]. The former were located covering the sheet-punch contact area and the latter in the rest of the sheet. This combination was successful to reproduce accurately the experimental strain evolutions measured by DIC, while avoiding locking phenomena. As it can be seen in Figure 7, a finer mesh was used in the sheet area in contact with the punch, where the simultaneous action of stretching and bending became significant.

The clamping by using a blankholder with drawbead (see Figure 3a) was simplified in the numerical model due to the time consuming requirements. This closure step was numerically modelled by a pre-strain of the sheet along the longitudinal axis and the subsequent pinned of the nodes in contact with the die. The pre-strain level was calibrated in accordance to the experimental data measured in the sheet via DIC after the complete closure of the blankholder. This assumption was successfully justified by comparing the stresses and strains evolutions during the entire experiment obtained with a full model (including the blankholder and drawbead) and with the simplified model, being both equivalents in the region of interest where failure occurred [61].

Regarding the material models, the metal sheet was considered to behave as an elastic-plastic rate-independent material. The mechanical properties are summarized in Table 1 and the plastic hardening law at 0° is given by Equation (1). The elastic behavior was supposed to be isotropic and the yield locus was described by the Barlat Yld’91 anisotropic criterion [62], as usual when modelling aluminum alloys [63,64,65]. The yield potential of this non-quadratic criterion is represented by:

(2) $$\Phi ={\left|{\tilde{S}}_{1}-{\tilde{S}}_{2}\right|}^{m}+{\left|{\tilde{S}}_{2}-{\tilde{S}}_{3}\right|}^{m}+{\left|{\tilde{S}}_{3}-{\tilde{S}}_{1}\right|}^{m}=2\sigma_{y}^{m}$$

where *m* is a constant related to the crystal lattice of the metal sheet, $\sigma_{y}$ is the yield stress and ${\tilde{S}}_{i}$ are the principal values of the transformed stress tensor $\tilde{S}=L\sigma$, which is a linear transformation (*L*) of the Cauchy stress tensor σ. Since the AA7075-O is a face-centered cubic (FCC) material, the *m* exponent was set to 8 [65,66]. Table 2 provides the anisotropy coefficients fitted from the *r*-values and the flow stress at 0°, 45° and 90° in the tensile tests. An isotropic and a kinematic hardening model, along with the Barlat Yld’91 yield function, were implemented.

The friction model followed a Coulomb’s law. The coefficients were set to μ = 0.05 in the punch-metal sheet contact and μ = 0.15 between die and metal sheet. These values were estimated by comparison of the experimental and numerical punch force vs. punch travel evolution from the Nakazima tests [61]. Similar values have been previously reported in literature for modelling thin-walled tube compression [67] and Erichsen cupping tests [68].

### 3.1. Accuracy of the Numerical Simulations

In order to verify the accuracy of the developed finite element model, experimental data measured via DIC from Nakazima and stretch-bending tests were verified with the numerical predictions.

**Figure 8:**
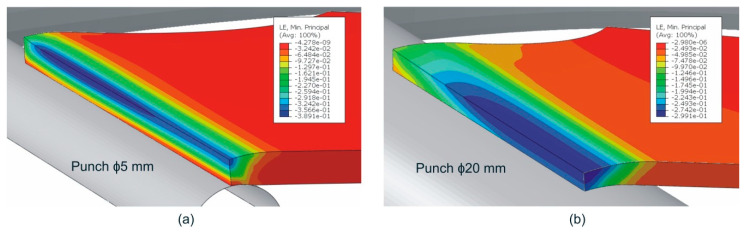
Numerical thickness strain contours in a stage near failure by necking for cylindrical punches of (**a**) ϕ5 mm and (**b**) ϕ20 mm (one quarter model).

Figure 8 shows the numerical thickness strain contours at the outer surface for cylindrical punches in a stage near failure by necking. For the sake of clarity, only the results for ϕ5 and ϕ20 mm punches are shown, being each one a representative case of severe and moderate strain gradient, respectively. As mentioned before the onset of local necking was experimentally observed using DIC at points located around a 75% of the specimen semi-width from the free-edge for the ϕ20 mm punch, a 25% for the ϕ10, ϕ5, and at 15% for ϕ3 and ϕ1 mm cylindrical punch tests. As shown in both figures, these locations successfully correspond to the regions in which the thickness reduction is concentrated in the simulations, i.e., where failure was expected. A similar agreement was also found for the rest of simulations.

**Figure 9:**
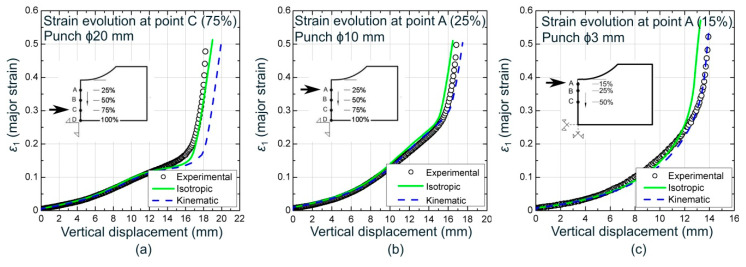
Experimental and numerical prediction of major strain at the outer surface versus vertical displacement for cylindrical punches of (**a**) ϕ20 mm, (**b**) ϕ10 mm, and (**c**) ϕ3 mm (one quarter model).

The evolutions of the major strain at the outer surface versus the vertical displacement at several points around the failure region for each case were also investigated. Figure 9 depicts three of these comparisons at points located at the 75%, 25%, and 15% of the specimen semi-width from the edge for the ϕ20, ϕ10, and ϕ3 mm cylindrical punches, respectively. Numerical predictions for both an isotropic and kinematic hardening model, along with the experimental data, are shown. As can be seen, the experimental data matched well with the numerical predictions using both models. Data from ϕ20 mm punch was better reproduced by using an isotropic model whereas the kinematic rule yielded accurate predictions for the ϕ10 mm and ϕ3 mm punches. It is worth noting that this last behavior was also observed for the rest of cylindrical punches simulations.

**Figure 10:**
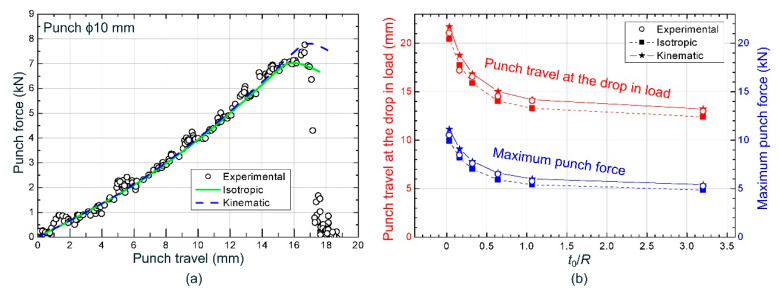
(**a**) Experimental punch force vs. punch travel for ϕ10 mm cylindrical punch; (**b**) maximum force and punch travel at the drop in load vs. *t*_0_/*R* for hemispherical (ϕ100 mm) and cylindrical punches (ranging from ϕ20 up to ϕ1 mm).

Finally, the evolutions of the punch force versus punch travel were also compared. As shown in Figure 10a for the ϕ10 mm cylindrical punch, the curve slope, the punch travel at the drop in load and the maximum punch force agreed well with experimental data. Figure 10b depicts the successful comparison of the numerical results of these two last variables with the experimental data for each punch diameter. As expected, the experimental data reasonably evolve between the isotropic and kinematic hardening models. Again, it seems that experimental trends are slightly better reproduced when a kinematic hardening model was used in the simulation, except for the ϕ20 mm cylindrical punch which is closer to a pure isotropic behavior.

In addition, as it will be discussed in the following section, stretch-bending processes undergo a reversal loading at the inner layers in the sheet thickness as a consequence of the simultaneous action of stretching and bending [1]. Therefore, a better agreement using a kinematic model was expected, pointing out its ability to reproduce the inverse plasticity that appears in stretch-bending controlled processes. According to this analysis, a pure kinematic hardening law was selected for assessing failure in the following sections.

## 4. Strain Analysis and Failure Model in Stretch-Bending

In a stretch-bending test, the punch performs a simple movement of translation (see for instance Figure 3a) at a predetermined speed. From the point of view of the external load, the punch force is applied in a proportional manner. However, this does not imply that all the material across the sheet thickness is subjected to proportional and even less uniform strain/stress field. In fact, there are through-thickness stresses in the sheet-punch contact area, stretching along the whole sheet and bending in the regions where the sheet is bent, i.e., in the contact areas with the punch and those adjacent to the die and blankholder corner radii. Thus, regions under stretching and bending are subjected to strain/stress gradients across the thickness, unlike what happens in pure stretching processes, causing the failure mechanism to be different.

**Figure 11:**
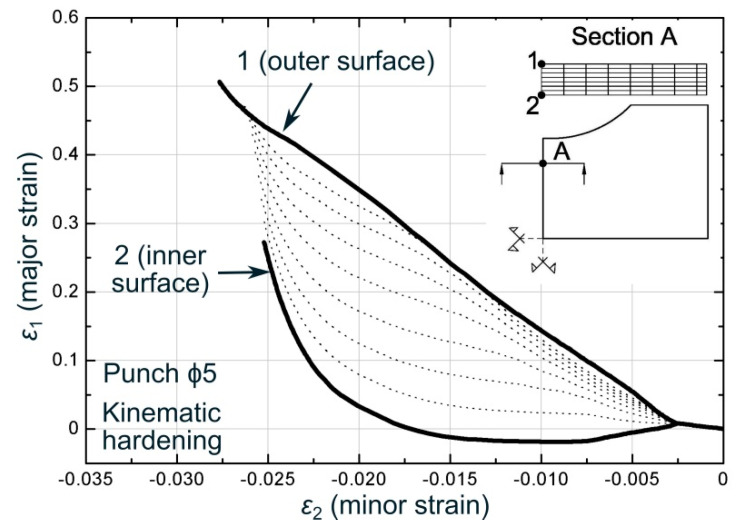
Strain path evolutions at the outer, intermediates, and inner surfaces for ϕ5 mm cylindrical punch around the failure location.

Figure 11 shows the numerical strain path evolutions, within the principal strain space, in a series of points through the sheet thickness located where failure occurs, that is, in a section at 25% of the specimen semi-width from the edge (Section A), for a stretch-bending test with a ϕ5 mm cylindrical punch. As it can be seen, in a first stage, corresponding to the blankholder closure, all the points across the sheet thickness evolved similarly. After that, when the punch started to move up, the strain paths became very different depending on the point analyzed. Thus, on the outer face (Point 1) the major strain was always positive and monotonously increasing, while on the inner surface (Point 2), that is, the one in contact with the punch, the major strain started decreasing and registering negative values as consequence of bending, and later it became positive when the stretching effect dominated the whole material cross-section, exhibiting therefore a reversal loading. The deformation process for the rest of the points across the thickness exhibited an intermediate evolution between those.

This evidence points out that the forming process induced a relevant non-uniform strain distribution (strain gradient) across the sheet thickness and the material, mainly the region near the inner surface, was subjected to a complex and clearly non-linear deformation history. Therefore, a reliable failure criterion in stretch-bending situations needs to take into account the existence of different states of strain, stress or any other variable controlling the sheet failure, in the sheet thickness due to the bending effect and also the use of strain path-independent metrics. Thus, in this research, the former is accomplished by recovering the concept of the critical distance rule (CDR), previously proposed and explored by the authors for assessing the influence of stress/strain gradients on the formability limits [26,29,30]. The latter is implemented by making use of limiting curves based on stress metrics, particularly the FLSC and epFLC, which seem to be apparently more insensitive to complex strain histories than the strain-based FLC [35,42].

### 4.1. Construction of the FLSC and epFLC

**Figure 12:**
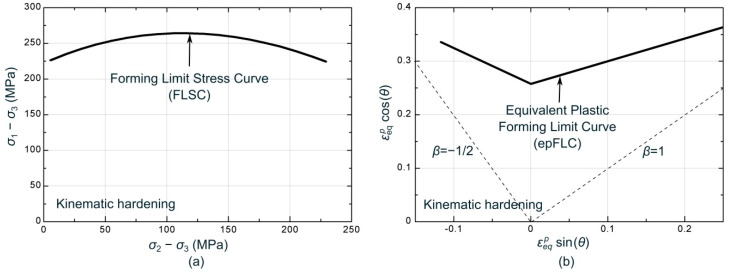
Numerically determined (**a**) FLSC and (**b**) epFLC based on the experimental FLC of the AA7075-O.

The strain-based FLC was numerically transformed into the stress-based metrics FLSC and epFLC respectively. The former was mapped within the $\sigma_{1}-\sigma_{3}$ vs. $\sigma_{2}-\sigma_{3}$ space and the latter into the $\varepsilon_{eq}^{p}\sin\left(\theta \right)\;\mathrm{vs}.\;\varepsilon_{eq}^{p}\cos\left(\theta \right)$ polar space, where θ represents the angle of the local strain ratio ($\beta =d\varepsilon_{2}/d\varepsilon_{1})$. Their evaluation was carried out by means of the previous numerical simulation of the Nakazima tests experimentally performed, using three different strain paths. For each simulation, the time instant in which any point located at the outer face intersects the experimental FLC, the onset of localized necking and the failure site are predicted. At this instant, the principal stresses, the effective strain and the local strain ratio were extracted for obtaining the limiting values for the FLSC and epFLC. As a result, the FLSC and epFLC determined from the experimental strain-based FLC are depicted in Figure 12.

### 4.2. Failure Prediction Based on the Critical Distance Rule (CDR)

Once the strain path-independent limiting curves and their key variables controlling the sheet failure have been defined, the next step for predicting sheet failure is to account the bending effect, i.e., the existence of non-uniform values of the variables across the thickness. For this regard, the use of the CDR, which is based on an extension of the critical distance concepts from fracture mechanics, seems to be plausible [26,28,29,30]. This rule recognizes that failure by necking under non-uniform strain/stress distributions is controlled by the accumulated damage in a certain material volume located at the inner side of the sheet, i.e., at the less strained/stressed material. This material volume is able to postpone the global instability through the whole thickness and can be characterized by a critical distance (**d_crit_**) measured from the inner surface, which depends on the material and its metallurgical properties. Thus, since all the material inside the critical volume contribute to postpone the plastic instability, it seems reasonable to assume that the failure is triggered by the average values of the key variables of the damage inside that volume when reaching the limiting condition.

Based on the above ideas, two different criteria exploding the CDR concept for stretch-bending are formulated as follows:

- The first alternative uses the principal stresses, particularly $\sigma_{1}-\sigma_{3}$ and $\sigma_{2}-\sigma_{3}$, as key variables for the sheet failure. Thus, it is postulated that the onset of localized necking begins when the average values of $\sigma_{1}-\sigma_{3}$ vs. $\sigma_{2}-\sigma_{3}$ at a certain critical distance (*d_crit_*) intersects the forming limit stress curve, FLSC. Hereafter, this failure model is referred as CDR-FLSC.
- The second variant makes use of the equivalent plastic strain ($\varepsilon_{eq}^{p}$) and the angle (θ) of the local strain ratio β within the polar diagram, defined by $\varepsilon_{eq}^{p}\sin\left(\theta \right)\;\mathrm{and}\;\varepsilon_{eq}^{p}\cos\left(\theta \right)$, as key variables. Failure by necking begins when the average values of the equivalent plastic strain within the polar diagram, at a predetermined *d_crit_*, reaches the polar epFLC. In the following, this failure model is named as CDR-epFLC.

## 5. Results and Discussion

The CDR-FLSC and CDR-epFLC models were applied to the finite element simulations of the stretch-bending tests performed over AA7075-O sheets using different cylindrical punches radii, from ϕ20 up to ϕ1 mm. Several values of critical distances, *d_crit_*, were explored and the numerical predictions of the necking strains, measured at the outer surface, were compared and discussed in the light of the experimental results.

### 5.1. Analysis of the Averaged Variables at Different Critical Distances within the Path-Independent Spaces

**Figure 13:**
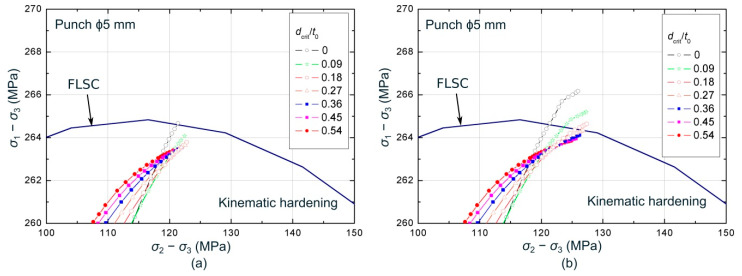
Numerical stresses evolutions, averaged at different values of *d_crit_*, for the ϕ5 mm stretch-bending tests at the time instants at which numerical paths for (**a**) *d_crit_* = 0 and for (**b**) *d_crit_* = 0.27 *t*_0_ reached the FLSC.

**Figure 14:**
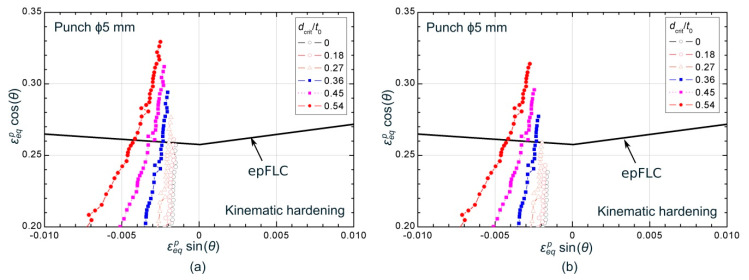
Equivalent plastic strain evolutions within the polar space, averaged at different values of *d_crit_*, for the ϕ5 mm stretch-bending tests at the time instants at which numerical paths for (**a**) *d_crit_* = 0 and for (**b**) *d_crit_* = 0.27 *t*_0_ achieved the epFLC.

Figure 13 and Figure 14 show the numerical evolutions, averaged at different values of *d_crit_*, within the $\sigma_{1}-\sigma_{3}$ vs. $\sigma_{2}-\sigma_{3}$ and $\varepsilon_{eq}^{p}\sin\left(\theta \right)\;\mathrm{vs}.{\;\varepsilon }_{eq}^{p}\cos\left(\theta \right)$ spaces, respectively, in a region close to the corresponding forming limiting curve, FLSC or epFLC, for the ϕ5 mm stretch-bending test. The critical distances are expressed as a percentage of the initial sheet thickness (*t*_0_), covering values from a 0% up to 54% of *t*_0_. The evolutions are shown until the time instant at which the numerical paths for a *d_crit_* = 0 (Figure 13a and Figure 14a) and for a *d_crit_* = 0.27 *t*_0_ (Figure 13b and Figure 14b) reached the limiting curve.

Regarding the stress space, it can be seen in Figure 13a that the evolution corresponding to *d_crit_* = 0 is the first in achieving the FLSC. As the critical distance increases, the stress evolutions take longer to reach the limit curve. In fact, Figure 13b, which shows the average stresses state when the numerical path at a *d_crit_* = 0.27 *t*_0_ reached the limiting condition, reveals that larger sizes of critical distance have not yet achieved the limiting curve, while smaller values have already exceeded it. Therefore, in this case, as the critical distance increases, the predicted limit strains will be greater. This trend was observed for tests performed using ϕ20, ϕ10, ϕ5, and ϕ3 mm punches. However, the cylindrical punch of ϕ1 mm showed the contrary trend, being the curve corresponding to *d_crit_* = 0 the last one in reaching the FLSC. In this case, the higher the critical distance, the smaller forming limits predicted. It should be highlighted that there are two competing factors that influence the order in which the curves, for each critical distance, reach the FLSC within the $\sigma_{1}-\sigma_{3}$ vs. $\sigma_{2}-\sigma_{3}$ space. On the one hand, the effect of the stress gradient imposed by the radius of the punch (bending effect), which generates higher values of in-plane stresses on the outer side than on the inner side. On the other hand, the effect of the contact pressure, which generates a maximum compressive stress *σ*_3_ at the inner face and zero at the outer layer. Therefore, the sequence in reaching the FLSC is established depending on which effect is dominant for each punch. In any case, it can be concluded from the stress values shown in Figure 13 that the range of stresses exhibited for this material is quite narrow, despite of the relevant strain gradient previously shown for the same test in Figure 11.

Within the polar space, Figure 14a shows that the evolution corresponding to a *d_crit_* = 0 was the last one in intersecting the epFLC. At that time instant, the paths for greater critical distances have already reached the failure condition, showing a contrary trend compared to those observed in the stress space for the same test. In addition, as can be seen in Figure 14b, this occurs in an orderly manner, that is, the first in reaching the limiting curve is those with greater critical distance and then, the others achieves the epFLC sequentially as the critical volume decreases. Thus, smaller critical distances will provide greater limit strains, as expected due to the bending effect. This behavior was exhibited for the complete series of cylindrical punches. On the other hand, it is worth noting that, within the polar space, a clear gradient across the sheet thickness in terms of equivalent plastic strain is observed, being consistent with the gradient observed in the principal strain space (see Figure 11 as reference).

### 5.2. Failure Prediction under Complex Deformation Histories

**Figure 15:**
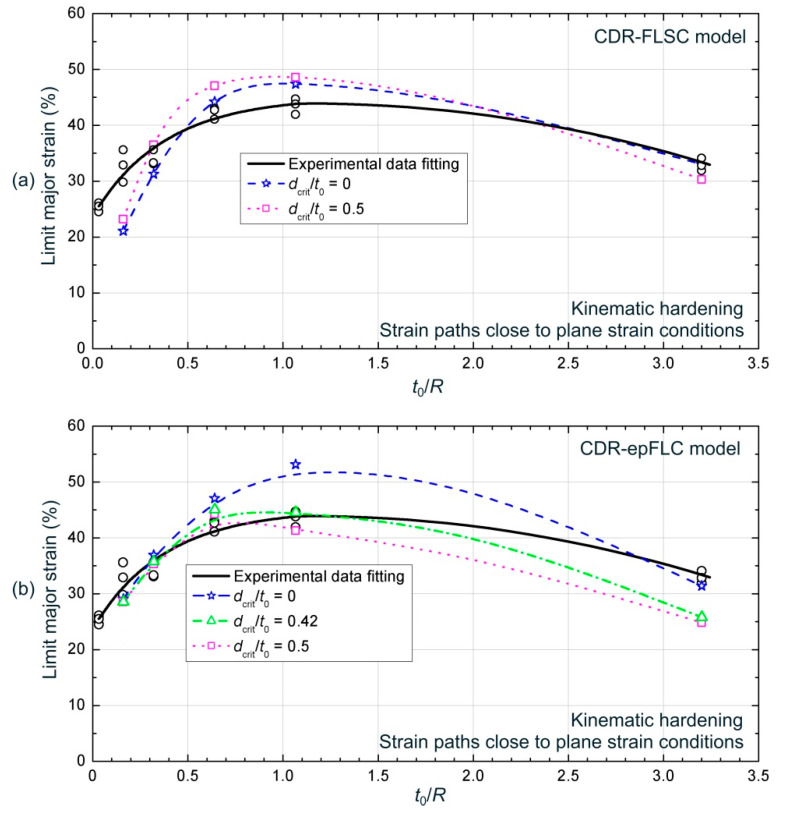
Experimental and numerical necking major strain (%) versus the *t*_0_/*R* ratio using (**a**) CDR-FLSC and (**b**) CDR-epFLC models for cylindrical punches.

Figure 15 depicts the predicted major strain at the onset of necking versus the bending ratio *t*_0_/*R* by using the CDR-FLSC and CDR-epFLC failure models, respectively, for the different cylindrical punch radii. For the sake of clarity, only the results for values of critical distance of *d_crit_* = 0 and half the thickness, *d_crit_* = 0.5 *t*_0_, are shown in the former, whereas it is also depicted the predictions for *d_crit_* = 0.42 *t*_0_ for the latter. The experimental data (black circles) and its fitting curve are also shown. Quantitative results of the predictions and the error with respect to the mean values of experimental data are shown in Table 3 for each case.

As can be observed in Figure 15a, CDR-FLSC criterion, for both critical distances, predicted limit strains for ϕ5 and ϕ3 mm slightly above the experimental data, whereas predictions for the ϕ20 mm punch underestimated the sheet formability. The numerical results for the other two punches, ϕ10 and ϕ1 mm, matched well with experimental values. As can be seen in Table 3a, predictions for ϕ10, ϕ5, ϕ3, and ϕ1 mm showed small deviations compared to the experimental data, less than 11%, providing a reasonably good agreement. The maximum deviation was observed for the ϕ20 mm punch. In this regard, it is worthy to note that the experimental results for this punch diameter yielded a relatively large scatter, as can be seen in Figure 5, and additionally, the agreement of the numerical model for this punch was more limited than for the others, as shown in Figure 9 and Figure 10. However, the most remarkable fact from Table 3a is that numerical predictions for both cases yielded similar results, despite of the substantially different values of critical distance, that is, the material seems to be not very sensitive to the size of the critical volume considered as responsible of the sheet failure. As previously mentioned, this apparent insensitivity with the critical distance is related with the existence of a smooth stress gradient ($\sigma_{1}-\sigma_{3}$ and $\sigma_{2}-\sigma_{3}$) across the sheet thickness at the onset of necking. In fact, in Figure 13, it can be seen that in the last stages close to the FLSC, the curves for different critical distances became very close, with differences of around 2–3 MPa. Thus, these averaged curves at different *d_crit_*, reached the FLSC at very close time instants, providing similar predictions in terms of strains. This smooth stress gradient, even when the material is subjected to a mild or severe strain gradient, is related to the Voce-type hardening response exhibited for the AA7075-O sheets. Anyway, although a little influence of the critical distance into the prediction were observed, in general, a slightly better results were provided with CDR-FLSC model by using a value of critical distance of half thickness ($d_{crit}=0.5\;t_{0}$), i.e., 800 µm.

Regarding the CDR-epFLC criterion, as can be seen in Figure 15b and Table 3b, the predicted results for ϕ20 and ϕ10 mm punches, that is, those that induced a smaller gradient, practically coincided with the experimental data for all the cases, although a slight improvement was observed for larger critical distances, i.e., *d_crit_* = 0.42 *t*_0_ and *d_crit_* = 0.5 *t*_0_. Instead, as the bending effect increased by using small radii punches, the beneficial role of increasing critical distance became crucial for obtaining accurate predictions. In fact, a value of *d_crit_* = 0 substantially overestimated the sheet formability for the ϕ5 and ϕ3 mm and slightly underestimated the limit strains for the ϕ1 mm, whereas sizes of critical volume of *d_crit_* = 0.42 *t*_0_ and *d_crit_* = 0.5 *t*_0_, also yielded results in very good agreement with the experimental formability for ϕ5 and ϕ3 mm punches, showing deviations below a 5%–6% compared to experimental data. Just the numerical prediction for the ϕ1 mm punch underestimated the experimental limit strains, probably due to the difficulties for reproducing the indentation process that experimentally occurred into the sheet. It should be noted that, as expected according to the analysis in Section 5.1, the predicted limit strains decreased as the critical distance increased for all the cases, showing a more relevant influence for the smaller punch radii.

In summary, except for the 1 mm punch due to indentation issues, the CDR-epFLC model provided more accurate and consistent predictions of the sheet formability by using a critical distance of around $0.42-0.5\;t_{0}$, i.e., 672–800 µm.

## 6. Conclusions

In this work, an analysis of the failure limits of stretch-bend AA7075-O sheets has been carried out. Firstly, the mechanical properties characterization and formability limits at necking under uniform and non-uniform strain distribution was performed. Secondly, by means of a calibrated 3D finite element model developed, the numerical predictions of forming limits in a series of stretch-bending tests were assessed and compared with the experimental results, using two different failure criteria based on the use of critical distance rule (CDR) and strain-path independent metrics. A summary of the main findings are as follows:

- The experimental major strains of necking increased with increasing *t*_0_/*R*, up to the ϕ3 mm punch, due to the beneficial effect of bending in the sheet failure. The enhancement of formability was around a 72% when using a ϕ3 mm punch compared to a Nakazima punch.
- The material in a stretch-bending process evolved under a complex deformation history, showing a reversal loading around the inner layers and non-uniform strain/stress distribution across the sheet thickness.
- A little influence of the critical distance into the predictions was observed for the CDR-FLSC model, due to the smooth stress gradient observed as consequence of the Voce-type hardening response exhibited for the AA7075-O sheets. In general, the model provided reasonably good results by using a value of critical distance of half thickness ($d_{crit}=0.5\;t_{0}$), i.e., 800 µm.
- The CDR-epFLC approach yielded the best predictions of the sheet formability at a critical distance of around $0.42-0.5\;t_{0}$, i.e., 672–800 µm. The beneficial effect of increasing critical distance became crucial for obtaining accurate predictions as the bending effect increased, that is, for the smaller punch radii.
- The results by CDR-epFLC were in better agreement with experimental data than the CDR-FLSC model for the whole range of bending ratio *t*_0_/*R*, except for the ϕ1 mm punch due to indentation issues.

As summary, for AA7075-O sheets of 1.6 mm thickness, failure in stretch-bending is triggered by a critical material volume of around the half thickness ($d_{crit}=0.42-0.5\;t_{0})$, measured from the inner surface, for the both path-independent stress-based metrics here analysed. These results provided a promising framework for predicting sheet failure by FEA of the manufacturing of industrial parts subjected to complex stretch-bend forming conditions.

## Figures and Tables

**Table 1:** Mechanical properties for AA7075-O sheets at 0°, 45° and 90°.

Orientation	*E* (GPa)	ν	*σ*_*Y*, 0.2%_ (MPa)	*UTS* (MPa)	*e_u_*	*r*
Rolling (0°)	68.1	0.3	102.3	203.0	0.149	0.812
Diagonal (45°)	68.1	0.3	102.4	208.0	0.178	1.394
Transverse (90°)	69.4	0.3	107.4	213.0	0.163	1.317

**Table 2:** Barlat’91 yield function coefficients for AA7075-O sheets.

*C*_1_	*C*_2_	*C*_3_	*C*_4_	*C*_5_	*C*_6_	*m*
0.926	1.021	0.978	1	1	1.007	8

**Table 3:** Comparison of experimental and numerical results of limit major strain at the outer surface using (**a**) CDR-FLSC and (**b**) CDR-epFLC models for different values of critical distances ($d_{crit})$.

**(a)**								
		**Experimental Data (mean)**	$\mathrm{Numerical}\;\mathrm{Predictions}\;\mathrm{CDR}\text{-}\mathrm{FLSC}\;d_{crit}=0$			$\mathrm{Numerical}\;\mathrm{Predictions}\;\mathrm{CDR}\text{-}\mathrm{FLSC}\;d_{crit}=0.5\;t_{0}\;$		
ϕ (mm)	*t*_0_/*R*	$\varepsilon_{1,lim}^{out}\left(exp\right)$	$\varepsilon_{1,lim}^{out}\;\left(pred\right)$		% Error*	$\varepsilon_{1,lim}^{out}\;\left(pred\right)$	% Error*	
20	0.16	0.328	0.211		−35.67	0.232	−29.27	
10	0.32	0.340	0.313		−7.94	0.365	7.35	
5	0.64	0.423	0.442		4.49	0.471	11.35	
3	1.07	0.435	0.474		8.97	0.486	11.72	
1	3.2	0.329	0.330		0.30	0.303	−7.90	
**(b)**								
		**Experim. Data (mean)**	**Numerical** **CDR-epFLC** $d_{crit}=0$		**Numerical CDR-epFLC** $d_{crit}=0.42t_{0}$		**Numerical CDR-epFLC** $d_{crit}=0.5t_{0}$	
ϕ (mm)	*t*_0_/*R*	$\varepsilon_{1,lim}^{out}\left(exp\right)$	$\varepsilon_{1,lim}^{out}\;\left(pred\right)$	% Error*	$\varepsilon_{1,lim}^{out}\;\left(pred\right)$	% Error*	$\varepsilon_{1,lim}^{out}\;\left(pred\right)$	% Error*
20	0.16	0.328	0.291	−11.28	0.286	−12.80	0.286	−12.80
10	0.32	0.340	0.369	8.53	0.357	5.00	0.353	3.82
5	0.64	0.423	0.471	11.35	0.450	6.38	0.443	4.73
3	1.07	0.435	0.532	22.30	0.445	2.30	0.413	−5.06
1	3.2	0.329	0.314	−4.56	0.258	−21.58	0.249	−24.32

* $\%Error=100\frac{\varepsilon_{1,lim}^{out}\;\left(pred\right)-\varepsilon_{1,lim}^{out}\left(exp\right)}{\varepsilon_{1,lim}^{out}\left(exp\right)}$

## References

[1] Uko D.K., Sowerby R., Duncan J.L. (1977). Strain distribution in the bending-under-tension test. CIM Bull..

[2] Keeler S.P., Backhofen W.A. (1963). Plastic instability and fracture in sheet stretched over rigid punches. Trans. ASM.

[3] ISO 12004-2:2008 (2008). Metallic Materials—Sheet and Strip—Determination of Forming-Limit Curves—Part 2: Determination of Forming-Limit curves in the Laboratory.

[4] Dicecco S., Butcher C., Worswick M., Boettcher E., Chu E., Shi C. (2016). Determination of forming limit diagrams of AA6013-T6 aluminum alloy sheet using a time and position dependent localized necking criterion. IOP Conf. Ser. Mater. Sci. Eng..

[5] Min J., Stoughton T.B., Carsley J.E., Lin J. (2017). Comparison of DIC methods of determining forming limit strains. Procedia Manuf..

[6] Song Y., Green D.E., Rose A. (2019). Investigation of various necking criteria for sheet metal formability analysis using digital image strain data. Int. J. Mater. Form..

[7] Sun L., Cai Z., He D., Li L. (2019). Aluminum alloy sheet-forming limit curve prediction based on original measured stress-strain data and its application in stretch-forming process. Metals.

[8] Jaremenko C., Ravikumar N., Affronti E., Merklein M., Maier A. (2019). Determination of forming limits in sheet metal forming using deep learning. Materials.

[9] Affronti E., Jaremenko C., Merklein M., Maier A. (2018). Analysis of forming limits in sheet metal forming with pattern recognition methods. Part 1: Characterization of onset of necking and expert evaluation. Materials.

[10] Jaremenko C., Affronti E., Maier A., Merklein M. (2018). Analysis of forming limits in sheet metal forming with pattern recognition methods. Part 2: Unsupervised methodology and application. Materials.

[11] Kleemola H.J., Pelkkikangas M.T. (1977). Effect of predeformation and strain path on the forming limits of steel, copper and brass. Sheet Metal. Ind..

[12] Borrego M., Morales-Palma D., Martínez-Donaire A.J., Centeno G., Vallellano C. (2019). Analysis of formability in conventional hole flanging of AA7075-O sheets: Punch edge radius effect and limitations of the FLC. Int. J. Mater. Form..

[13] Charpentier P. (1975). Influence of punch curvature on the stretching limits of sheet steel. Met. Mater. Trans. A.

[14] Raghavan K.S. (1995). A simple technique to generate in-plane forming limit curves and selected applications. Met. Mater. Trans. A.

[15] Moshksar M.M., Mansorzadeh S. (2003). Determination of the forming limit diagram for Al 31055 sheet. J. Mater. Process. Tech..

[16] Fictorie E., Van den Boogaard A.H., Atzema E.H. (2010). Influence of punch radius in a Nakazima test for mild steel and aluminium. Int. J. Mater. Form..

[17] Affronti E., Merklein M. (2018). Analysis of the bending effects and the biaxial pre-straining in sheet metal stretch forming processes for the determination of the forming limits. Int. J. Mech. Sci..

[18] Neuhauser F.M., Terrazas O.R., Manopulo N., Hora P., Van Tyne C.J. (2016). Stretch bending—The plane within the sheet where strains reach the forming limit curve. IOP Conf. Ser. Mater. Sci. Eng..

[19] Neuhauser F.M., Terrazas O., Manopulo N., Hora P., Van Tyne C. (2019). The bending dependency of forming limit diagrams. Int. J. Mater. Form..

[20] Tharrett M.R., Stoughton T.B. (2003). Stretch-Bend Forming Limits of 1008 AK Steel.

[21] Kitting D., Koplenig M., Ofenheimer A., Pauli H., Till E.T. (2009). Application of a “concave-side rule” approach for assessing formability of stretch-bent steel sheets. Int. J. Mater. Form..

[22] Martínez-Donaire A.J., Vallellano C., Morales-Palma D., García-Lomas F.J. (2012). Experimental and numerical analysis of the failure of AA7075-O stretch-bend sheets. Steel Res. Int..

[23] Martínez-Donaire A.J., Martínez-Palmeth L.H., Borrego M., Morales-Palma D., Vallellano C. (2019). Analysis of the failure of H240LA steel sheets subjected to strecth-bending conditions. Proc. Manuf..

[24] Wu J., Zhou D., Zhang L., Zhou Y.J., Du C.Q., Shi M.F. (2006). A failure criterion for stretch bendability of advanced high-strength steels. SAE Trans..

[25] Sadagopan S., Wong C., Huang M., Yan B. (2003). Stretch bendability of advanced high-strength steels. SAE Paper.

[26] Vallellano C., Morales-Palma D., Martínez-Donaire A.J., García-Lomas F.J. (2010). On the use of Concave-Side Rule and Critical-Distance Methods to predict the influence of bending on sheet-metal formability. Int. J. Mater. Form..

[27] Taylor D. (2007). The Theory of Critical Distances: A New Perspective in Fracture Mechanics.

[28] Morales-Palma D., Vallellano C., García-Lomas F.J. (2013). Assessment of the effect of the through-thickness strain/stress gradient on the formability of stretch-bend metal sheets. Mater. Des..

[29] Morales-Palma D., Martínez-Donaire A.J., Vallellano C. (2017). On the use of maximum force criteria to predict localised necking in metal sheets under stretch-bending. Metals.

[30] Martínez-Palmeth L.H., Martínez-Donaire A.J., Vallellano C. (2019). Formability limits of high-strength H240LA steel sheets under stress/strain gradients. Mech. Mater..

[31] Graf A.F., Hosford W.F. (1993). Effect of changing strain paths on forming limit diagrams of Al 2008-T4. Met. Trans. A.

[32] Arrieux R. (1987). Determination of the forming limit stress curve for anisotropic sheets. Ann. CIRP.

[33] Arrieux R. (1995). Determination and use of the forming limit stress diagrams in sheet metal forming. J. Mater. Process. Technol..

[34] Stoughton T.B. (2000). A general forming limit criterion for sheet metal forming. Int. J. Mech. Sci..

[35] Stoughton T.B., Yoon J.W. (2011). A new approach for failure criterion for sheet metals. Int. J. Plast..

[36] Bandyopadhyay K., Basak S., Panda S.K., Saha P. (2015). Use of stress based forming limit diagram to predict formability in two-stage forming of tailor welded blanks. Mater. Des..

[37] Panich S., Liewald M., Uthaisangsuk V. (2018). Stress and strain based fracture forming limit curves for advanced high strength steel sheet. Int. J. Mater. Form..

[38] Hakoyama C.S., Hakoyama T., Fukiharu H., Kuwabara T. (2019). Fracture prediction for mild steel sheet and high-strength steel sheet subjected to draw bending using forming limit stress criterion. J. Mater. Process. Technol..

[39] Huang T., Zhan M., Wang K., Chen F., Guo J., Li Y., Song Z., Bai L. (2019). Forming limit stress diagram prediction of pure titanium sheet based on GTN model. Materials.

[40] Yoshida K., Kuwabara T. (2007). Effect of strain hardening behaviour on forming limit stresses of steel tube subjected to non proportional loading paths. Int. J. Plast..

[41] Simha C.H.M., Gholipour J., Bardelcik A., Worswick M.J. (2007). Prediction of necking in tubular hydroforming using an extended stress-based forming limit curve. J. Eng. Mater. Technol..

[42] Stoughton T.B., Yoon J.W. (2012). Path independent forming limits in strain and stress spaces. Int. J. Solids Struct..

[43] Nguyen N.T., Lee E., Lee M.G., Kim H.J., Kim H.Y. (2015). Hydroformability assessment of AA6063 tubes using the polar effective plastic strain diagram. Proc. Inst. Mech. Eng. Part B J. Eng. Manuf..

[44] ASTM E8/E8M-08 (2008). Standard Test Methods for Tension Testing of Metallic Materials.

[45] ASTM E132-04 (2004). Standard Test Method for Poisson’s Ratio at Room Temperature.

[46] ASTM E517-00 (2000). Standard Test Method for Plastic Strain Ratio r for Sheet Metal.

[47] Vacher P., Haddad A., Arrieux R. (1999). Determination of the forming limit diagrams using image analysis by the correlation method. CIRP Ann. Manuf. Tech..

[48] Pan B., Qian K., Xie H., Asundi A. (2009). Two-dimensional digital image correlation for in-plane displacement and strain measurement: A review. Meas. Sci. Technol..

[49] Orteu J.J. (2009). 3-D computer vision in experimental mechanics. Opt. Lasers Eng..

[50] Jin T.L., Ha N.S., Goo N.S. (2014). A study of the thermal buckling behavior of a circular aluminum plate using the digital image correlation technique and finite element analysis. Thin-Walled Struct..

[51] Martínez-Palmeth L.H., Martínez-Donaire A.J., Centeno G., García-Lomas F.J., Vallellano C. (2013). Formability of automotive H240LA steel sheets in stretch-bending processes. Procedia Eng..

[52] Gorszczyk J., Malicki K., Zych T. (2019). Application of digital image correlation (DIC) method for road material testing. Materials.

[53] Mora-Macías J., Ayensa-Jiménez J., Reina-Romo E., Doweidar M.H., Domínguez J., Doblaré M., Sanz-Herrera J.A. (2020). A multiscale data-driven approach for bone tissue biomechanics. Comput. Methods Appl. Mech. Eng..

[54] Jain M., Lloyd D.J., MacEwen S.R. (1996). Hardening laws, surface roughness and biaxial tensile limit strains of sheet aluminium alloys. Int. J. Mech. Sci..

[55] Butuc M.C., Gracio J.J., Barata da Rocha A. (2003). Theoretical study on forming limit diagrams prediction. J. Mater. Process. Technol..

[56] Li J., Carsley J.E., Stoughton T.B., Hector L.G., Hu S.J. (2013). Forming limit analysis for two-stage forming of 5182-O aluminum sheet with intermediate annealing. Int. J. Plast..

[57] Martínez-Donaire A.J., Vallellano C., Morales D., García-Lomas F.J. (2010). Experimental detection of necking in stretch-bending conditions, a critical review and new methodology. Steel Res. Int..

[58] Martínez-Donaire A.J., García-Lomas F.J., Vallellano C. (2014). New approaches to detect the onset of localised necking in sheets under through-thickness strain gradients. Mater. Des..

[59] Silva M.B., Martínez-Donaire A.J., Centeno G., Morales-Palma D., Vallellano C., Martins P.A.F. (2015). Recent approaches for the determination of forming limits by necking and fracture in sheet metal forming. Procedia Eng..

[60] Centeno G., Martínez-Donaire A.J., Morales-Palma D., Vallellano C., Silva M.B., Martins P.A.F., Davim J.P. (2016). Novel experimental techniques for the determination of the forming limits at necking and fracture. Materials Forming and Machining: Research and Development.

[61] Martínez-Donaire A.J. (2012). Análisis del Efecto del Gradiente de Deformaciones en el Conformado de Chapas Metálica. Ph.D. Thesis.

[62] Barlat F., Lege D.J., Brem J.C. (1991). A six-component yield function for anisotropic metals. Int. J. Plast..

[63] Habraken F.A.C.M., Dautzenberg J.H. (1995). Some applications of the Barlat 1991 yield criterion. CIRP Ann. Manuf. Technol..

[64] Barlat F., Becker R.C., Hayashida Y., Maeda Y., Yanagawa M., Chung K., Brem J.C., Lege D.J., Matsui K., Murtha S.J. (1997). Yielding description for solution strengthened aluminum alloys. Int. J. Plast..

[65] Wu P.D., Jain M., Savoie J., MacEwen S.R., Tuğcu P., Neale K.W. (2003). Evaluation of anisotropic yield functions for aluminum sheets. Int. J. Plast..

[66] Zhang F., Chen J., Chen J., Zhu X. (2014). Forming limits model evaluation for anisotropic sheet metals under through-thickness normal stress. Int. J. Mech. Sci..

[67] Ha N.S., Lu G., Xiang X. (2018). High energy absorption efficiency of thin-walled conical corrugation tubes mimicking coconut tree configuration. Int. J. Mech. Sci..

[68] Vallellano C., Morales-Palma D., García-Lomas F.J. (2008). A study to predict failure in biaxially stretched sheets of aluminum alloy 2024-T3. Mater. Manuf. Process..

